# Hidradenitis Suppurativa and 1-Carbon Metabolism: Role of Gut Microbiome, Matrix Metalloproteinases, and Hyperhomocysteinemia

**DOI:** 10.3389/fimmu.2020.01730

**Published:** 2020-08-19

**Authors:** Jack Molnar, Carissa Jo Mallonee, Dragana Stanisic, Rubens P. Homme, Akash K. George, Mahavir Singh, Suresh C. Tyagi

**Affiliations:** ^1^Department of Physiology, University of Louisville School of Medicine, Louisville, KY, United States; ^2^Department of Dentistry, Faculty of Medical Sciences, University of Kragujevac, Kragujevac, Serbia

**Keywords:** acne, dysbiosis, extracellular matrix, homocysteine, tissue remodeling

## Abstract

Hidradenitis suppurativa (HS) is a chronic, inflammatory skin condition characterized by painful nodules which suppurate and later develop into scar tissues followed by the development of hypodermal tracts. Although the mechanisms behind HS are not fully understood, it is known that dietary factors play important roles in flare frequency and severity. We hypothesize that the high fat diet (HFD) causes dysbiosis, systemic inflammation, and hyperhomocysteinemia (HHcy) in susceptible individuals, which subsequently elevate inflammatory cytokines such as IL-1β, IL-6, IL-17, and tumor necrosis factor alpha (TNF-α). This increase in dysbiosis-led inflammation coupled with a dysregulation of the 1-carbon metabolism results in an increase in matrix metalloproteinases MMP-2, MMP-8, and MMP-9 along with tissue matrix remodeling in the development and maintenance of the lesions and tracts. This manuscript weaves together the potential roles played by the gut microbiome, HHcy, MMPs, and the 1-carbon metabolism toward HS disease causation in susceptible individuals.

## Introduction

Hidradenitis Suppurativa (HS), also known as acne inversa (AI), is a chronic, inflammatory skin condition caused by occlusion of the apocrine sweat glands (1). The Disease is characterized by painful nodules in the axillary, inguinal, and perianal regions, where the apocrine sweat gland density is highest (2). These nodules swell, rupture or suppurate, and release pus. The resulting lesions are painful and develop into scar tissue. Over time, tracts will develop and invade new areas. HS is painful, disfiguring, and in some cases, can be debilitating. The exact cause of the disease is currently unknown. Many aggravating factors may exacerbate the condition, including obesity (3), sex steroids (4), immune disorders (5), smoking, and hyperhomocysteinemia (HHcy). Since HS is considered an inflammatory disease and homocysteine (Hcy) is directly involved in inflammatory processes, and interestingly the data confirm that HHcy is more prevalent among patients with HS, acne vulgaris, and psoriasis. Therefore, evaluation of Hcy plasmatic levels should be advisable in HS patients (6). Of these factors, obesity, and smoking (7) may have the highest impact on disease progression, and severity. Bacterial flora are normal in early lesions, indicating that if infection is present, it is secondary (8). Previous research suggests that the chronic nature of the inflammation is due to the persistence of Biofilms (9). Further, HS is associated with many comorbidities such as inflammatory bowel disease (IBD) (10), metabolic syndrome (11), and hyperandrogenism, although the mechanisms underlying these associations are mostly unknown. HS is inherited with an autosomal dominant inheritance pattern (12), but the clinical presentation features a 3:1 female to male ratio in populations of European and African descent. It is unclear why this may be. HS is much less common in East Asian (13) populations where the ratio is paradoxically flipped with a 1:2.5 female to male ratio. Treatment of HS traditionally includes either surgery to excise the affected areas, deroofing, or the use of antibiotics such as rifampicin and clindamycin (14), although it is unknown whether the effect is due to the bacteriostatic or anti-inflammatory properties of these drugs. While these treatments are still in use today, novel approaches may offer relief to HS sufferers. Among these, laser hair removal can be effective. Further, TNF inhibitors such as adalimumab (15) also improve symptoms.

It has long been known that diet and the health of the gut play an important role in the health of the patient. Diets rich in fat and carbohydrates or low in fiber have all been linked to gut dysbiosis. Diets that include food from a variety of sources, including foods rich in plant pigments (16), are protective against dysbiosis. While a Western diet that is high in fats may be tolerable for some people, it can exacerbate underlying problems in others. In this manuscript, we outline the role of a high fat diet (HFD) in dysbiosis, which leads to systemic inflammation that exacerbates the HS condition through activation of Matrix Metalloproteases (MMP) along with the role of 1-carbon metabolism.

## High Fat Diet Leads to a Shift in Microbiome Composition and Inflammation

It has long been postulated that the diet likely plays a significant role in the pathogenesis of HS, but what is that role? A HFD is known to cause dysbiosis in the gut, leading to an increase in the ratio of Firmicutes to Bacteroidetes. The mechanism for this phenomenon is outlined by Guo et al., who found that in mice, a high fat diet leads to a decrease in the release of antimicrobial peptides in the small intestine, which is followed by changes in the composition of the gut microbiota, followed by alterations in the level of serum inflammatory cytokines (**Figure 2**) (17). Guo et al. fed C57BL/6J mice a high fat diet (60% of total calories) and sacrificed at 8, 12, or 16 weeks. Real-time PCR performed on the intestinal contents revealed that at the phylum level, Firmicutes were elevated and Bacteroidetes were decreased, compared to control at 8 weeks and there were no further changes at 12 or 16 weeks. As for genera, the composition of microflora had similarly changed by 8 weeks and saw little change at weeks 12 and 16. The authors also performed RT- PCR with small intestine epithelia for antimicrobial peptides. Expression of lysozyme, angiogenin 4, and Reg IIIγ decreased at 8, 12, and 16 weeks. Immunohistochemistry and Western blotting measured lysozyme expression, which decreased by 16 weeks. The authors also examined the inflammatory cytokines TNF-α, IFN- γ, IL-1β, and IL-6. Except for IFN-γ, all other inflammatory cytokines were upregulated. Lastly, circulating inflammatory cytokines IFN- γ, IL-1β, IL-6, IL-2, IL-10, and TNF-α were measured in serum. Oddly, IL-6 was absent and little change was seen at 8 and 12 weeks. However, at 16 weeks, IFN-γ and TNF-α were significantly increased.

In addition to small intestine antimicrobial peptides, the colonic mucus layer provides an essential innate defense against harmful bacteria. The Western diet is typically high in fats and simple sugars while low in dietary fiber. Birchenough et al. (18) found that this reduction in dietary fiber decreases production of the mucus barrier, which otherwise separates the microbiota from the gut epithelium. Reduction of the mucosal barrier is associated with an increase in epithelial inflammation.

## High Fat Diet, 1-Carbon Metabolism, and HS

Hwang et al. (19) fed C57BL/6 mice a high fat diet consisting of 60% fat. After 5 weeks, the mice had unsurprisingly gained weight and developed fatty livers with microscopically evident lipid vacuoles. PCR demonstrated significant upregulation of cystathionine-beta-synthase (CBS) and cystathionine-gamma-lyase (CSE). These transsulfuration pathway enzymes are responsible for converting homocysteine to hydrogen sulfide. As a result, homocysteine levels were lower in HFD mice compared to control.

To date, little research has been conducted on the effects of hyperhomocysteinemia (HHcy) in HS. Researchers have found elevated Homocysteine (Hcy) levels in the blood plasma of HS patients (6, 20). Marasca et al. (21) found that not only are Hcy levels elevated in HS patients, but the Hcy levels are positively correlated with disease severity, measured by Sartorius Score. The Sartorius Score is a measure of disease severity in HS which evaluates patients based on a point system (22). Because a HFD decreases circulating Hcy, there must be another mechanism responsible for HHcy in HS patients.

## MMP-8 in HS Skin Biopsies, Blood-Born Neutrophils, and Cultured Skin Cells

Tsaousi et al. (23) found that MMP-8 is upregulated in HS lesions (23). The authors obtained biopsies from surgically removed skin of HS patients and these biopsies were used for immunohistochemical staining. A total of 25 related molecules were examined, including many pro-inflammatory cytokines, and MMP-8. Neutrophilic granulocytes in HS lesions secreted high amounts of MMP-8 after stimulation by TNF-α. Fibroblasts expressed MMP-8 but not keratinocytes. The authors also found high levels of MMP-8 in the blood after stimulation with TNF-α. This finding in the blood also correlated to Sartorius score and severity of disease. The authors also obtained and stimulated blood samples with IFN-γ, TNF-α, IL-6, IL-17A, or IL-22 to activate neutrophils. In the blood of HS patients stimulated by TNF-α, they found a significant increase in the expression of MMP-8 and this increase was positively correlated with disease severity. There was no significant increase in the production of tissue inhibitor of metalloproteinases 4 (TIMP-4). Dermal fibroblasts and epidermal keratinocytes were cultured as well. Fibroblasts were stimulated using IFN-γ, TNF-α, IL-6, IL-17A, IL-19, or IL-24. Keratinocytes were either stimulated with TNF-α, IL-17A, or IL-22 or combinations of these cytokines. The dermal fibroblasts increased MMP-8 production when exposed to TNF-α, but the epidermal keratinocytes did not respond to TNF-α, IL-17A, or IL-22.

## MMP-2 is Upregulated in HS Lesional Skin

Mozeika et al. (24) carried out a study involving 14 Caucasian patients with HS. Tissue sections were prepared from paraffin-embedded tissues. The authors examined the expression of the antimicrobial peptide human beta defensin 2 (HBD-2), TNF-α, and MMP-2 in samples collected from the HS and volunteer groups through immunohistochemistry. In HS affected skin, HBD-2 was negative in 12 of the 14 samples. MMP-2 was found in keratinocytes, macrophages, and lymphocytes, fibroblasts, sweat glands, the outer epithelial sheath of hair follicles, and sinus tracts. TNF-α positive macrophages and lymphocytes were found in the dermis and the number varied from a few cells to many, correlated to the degree of disease severity. In the negative control group, TNF-α positive cells were not present. However, a moderate number of HBD-2 and MMP-2 positive structures were observed in the epithelium, sub-epithelium, and sweat glands.

## MMP-2 and MMP-9 in Cultured Biopsies of Lesional and Peri-Lesional Skin

Recently, Sanchez et al. produced an *ex-vivo* cultured skin model for HS (25) and observed the specimens through immunohistochemistry. MMP-2 and MMP-9 were elevated in lesional skin. IL-1β was found in all skin samples, although it was higher in lesional samples. The Hurley clinical staging system (Stage I is a single lesion without sinus tract formation, Stage II manifests as more than one lesion or area, but with limited tunneling and Stage III with multiple lesions), measures disease progression based on the number of nodules or abscesses, the number of body locations involved, and the presence or absence of tracts (26). There were 9 HS patients classified by Hurley stage (one patient in Stage 1, five patients in stage 2, and three patients in stage 3). In HS skin, biopsies were taken from lesions and peri-lesional sites. These samples were cultured and examined either at day 0 or day 4, then fixed, embedded in paraffin, and sectioned for microscopy. The authors looked for changes in collagen structure and infiltration. A second set of skin explants was used to prepare skin homogenate to examine cytokine concentration, MMP levels, and stimulation of keratinocytes. Gel zymography was performed with cell culture supernatants. Compared with controls, HS skin cultures maintained the skin architecture. No significant difference in the organization of type 1 and type 3 Collagen fibers was observed in the papillary or reticular dermis. However, degradation of elastin was observed after 4 days in lesional skin. At day 0, peri-lesional and control skin showed similar levels of inactive zymogen MMP-2 and MMP-9 expression, while lesional skin produced high levels of both inactive and active MMP-2 and MMP-9. At day 4, MMP-2 and MMP-9 secretion from peri-lesional skin was increased. Homogenized samples at day 0 and day 4 were also taken. At day 0, IL-1β concentration was elevated in HS lesional skin compared with peri-lesional and control skin. After 4 days, IL-1β was expressed in all samples but remained significantly higher in HS lesional skin compared to control. Concentrations of IL-17 and the inflammasome components NLRP3 and Caspase-1 were similar between all groups.

## MMP-2 and MMP-9 in Pyoderma Gangrenosum, Acne, and Suppurative Hidradenitis

Pyoderma gangrenosum (PG) is a chronic inflammatory skin condition that results in skin ulcers often found on the legs or hands. Pyoderma gangrenosum, acne, and suppurative hidradenitis (PASH) is the syndromic form of PG. Marzano et al. sought to examine similarities between PG and PASH as they relate to cytokine and MMP expression (27). The authors recruited 13 patients diagnosed with PG, 7 PASH diagnosed with stage 3 HS according to Hurley staging, and 6 volunteers as healthy skin controls. Biopsies were taken from lesional skin, samples were homogenized, and cell lysate was extracted for analysis. Cytokine antibody arrays were used to examine the expression of TNF-α, IL-17, IL-1β, and the receptors for each of these cytokines. Other proteins examined included leukocyte selectin (L-selectin), epithelial selectin (E-selectin), IL-8, regulated on activation normal T-cell expressed and secreted (RANTES), chemokine CXC-motif ligand 1/2/3 (CXCL 1/2/3), CXCL 16, MMP-2, MMP-9, TIMP-1, and TIMP-2. The authors found significant overexpression of TNF-α, IL-17, IL-1β, and their receptors. The lesional skin of both PG and PASH groups overexpressed L-selectin, E-selectin, IL-8, CXCL 16, and RANTES. Expression of MMP-2, MMP-9, TIMP-1, and TIMP-2 was significantly higher in both PG and PASH groups as well.

## Conclusion

**Figure 1:**
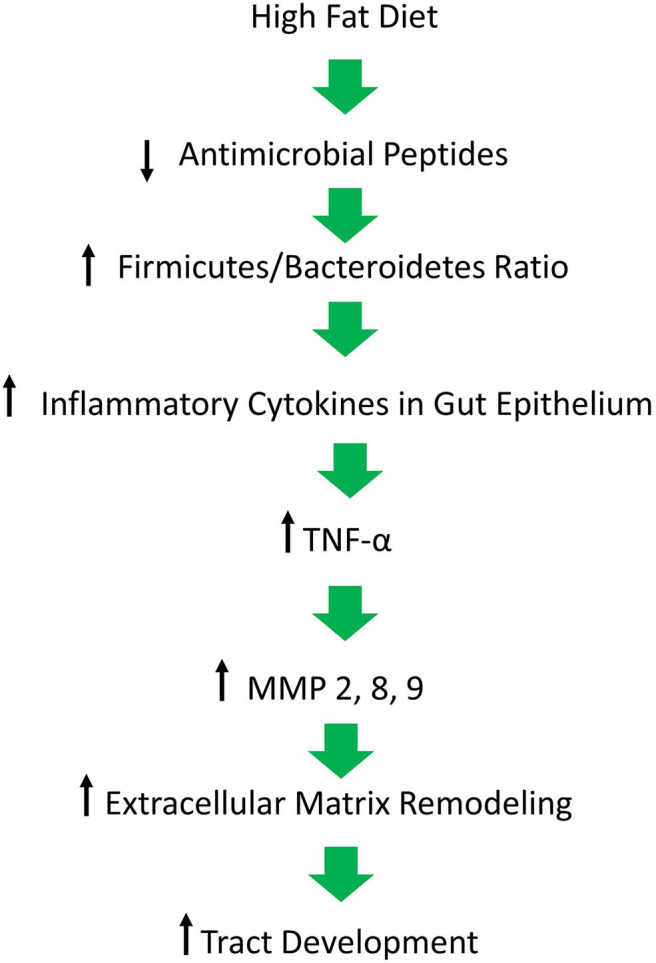
Figure depicting our hypothesis for the molecular mechanisms responsible for the inflammatory milieu that leads to continued inflammation in lesions and the development of tracts. A high fat diet causes a decrease in antimicrobial peptides which is followed by a pathological shift in the composition of the gut microbiome and an increase in inflammation, ultimately resulting in matrix remodeling and tract formation in the skin of affected individuals.

**Figure 2:**
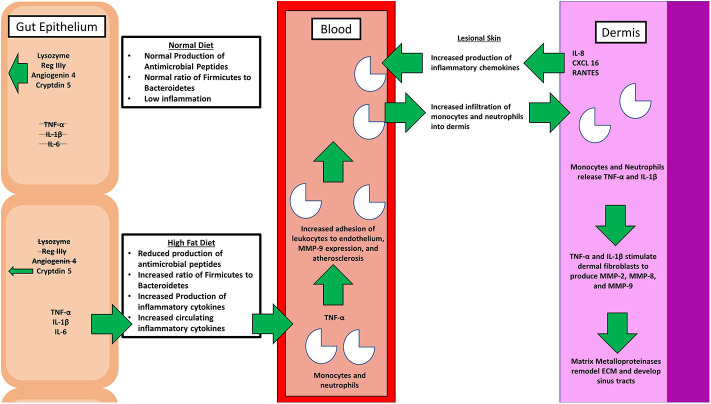
A simple schematic comparing the physiological effects of a normal diet with that of the pathological effects of a high fat diet (HFD). A healthy diet promotes the production of antimicrobial peptides, and proper balance of the microbiome genera. On the contrary, a poor diet affects the intimate relationship between gut microbiota, inflammation, and hidradenitis suppurativa (HS). Various inflammatory cytokines, including tumor necrosis factor alpha (TNF-α) and interleukin 1 beta (IL-1β), along with matrix metalloproteinases (MMPs) that are involved in the pathogenesis are depicted.

**Figure 3:**
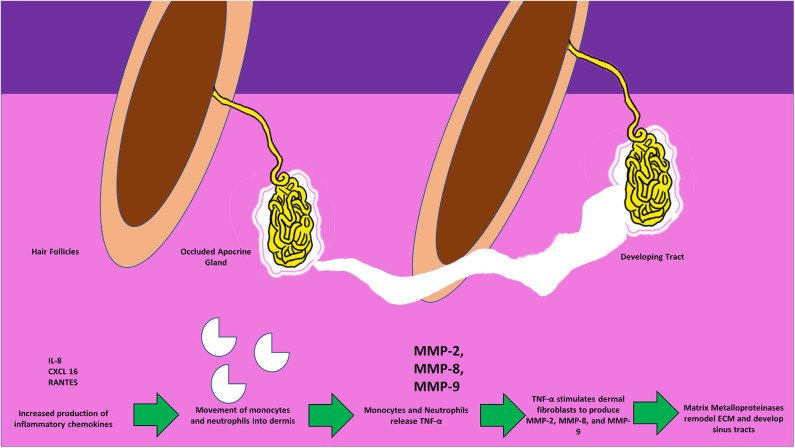
Figure depicting the invasion of monocytes, neutrophils, the release of active MMPs, and the development of sinus-tracts in the dermis in response to the surging inflammatory chemokines. Circulating TNF-α further stimulates dermal fibroblasts to produce MMPs thus causing the spread of tracts, and invasion of the surrounding new areas.

**Figure 4:**
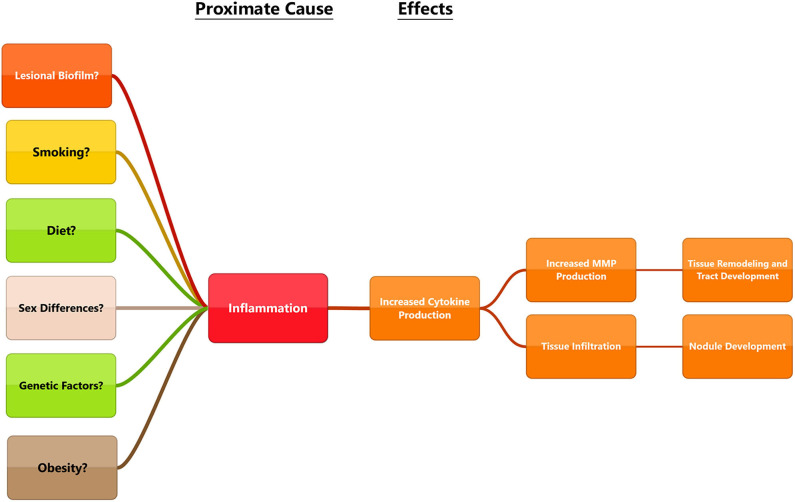
Diagram outlining some of the proposed ultimate causes in the pathogenesis of HS and connecting them to proximate causes. While each of these factors can lead to be inflammation, disease triggers may be different for everyone. Multiple factors may influence the disease and a holistic view will be necessary to find solutions that work for patients.

Beginning with the gut, we see an association between the HFD and an early reduction in antimicrobial peptides (Figure 1). An increase in the number of Firmicutes relative to Bacteroidetes follows alterations in the innate defenses of the gut (Figure 2). Other studies have linked similar shifts in the composition of the gut microbiome to irritable bowel syndrome and a host of other ailments (28). This realignment of intestinal flora coincides with an increase in the production of inflammatory cytokines such as TNF-α, IL-1β, and IL-6 by the intestinal epithelia which is followed by an increase in circulating inflammatory cytokines namely IFN-γ, and TNF-α. Paradoxically, a HFD lowers serum homocysteine while HHcy and HFD are linked to disease severity in HS patients, indicating a possible deficiency of the enzymes CSE and CBS. The HFD also leads to an increase in intestinal permeability, and an increase in circulating LPS, and other inflammation-stimulating molecules (29). Circulating TNF-α aids in the infiltration of neutrophils (30). Further, the overexpression of chemokines IL-8, CXCL 16, and RANTES as well as L-selectin, and E-selectin in PG/PASH lesional skin may also lead to the increased neutrophil and monocyte infiltration (30) in HS lesional skin. After infiltration of the skin, these leukocytes release cytokines, particularly IL-1β and TNF-α (23, 31), which result in increased expression of MMPs. It is well-established that inflammation mediated by TNF-α can increase the expression of MMPs (32) and these MMPs are responsible for matrix remodeling. Increased production of MMP-2, MMP-8, and MMP-9 are associated with HS lesional skin, and tract formation through matrix remodeling (Figure 3). The production of MMP-2 and MMP-8 are positively correlated with disease severity according to Hurley stages and Sartorius score, while disease severity was not considered in the examination of MMP-9. MMPs would likely be required for the massive tissue remodeling involved in the development of the hypodermal tracts in the progression of HS (Figures 1–3). Pyoderma gangrenosum and PASH HS lesions also exhibit overexpression of MMP-2 and MMP-9. The factors that cause HS have not yet been fully elucidated because they appear to be many and disparate (Figure 4). Solving HS is a problem much larger than identifying a single gene, pathogen, or a food item. Innumerous factors increase the frequency of lesions as well as the severity of the disease. To complicate matters further, HS is associated with a plethora of comorbidities that include dysregulation of the 1-carbon metabolism (6, 20, 21, 33–35). This ambiguity has led to the suggestion of many ultimate causes. The potential role of inflammatory pathway(s) as a proximate cause is clear. Thus, future work should be dedicated to finding the link between aggravating factors, such as diet and smoking, and inflammation, cytokine production, and MMP-mediated tissue remodeling, as depicted in Figure 3.

## Author Contributions

The authors confirm that they are the original contributors of this work and all of them approved it for its publication.

## Conflict of Interest

The authors declare that the research was conducted in the absence of any commercial or financial relationships that could be construed as a potential conflict of interest.

## Abbreviations

AI: acne inversa
CXCL: chemokine CXC-motif ligand
CSE: cystathionine-γ-lyase
CBS: cystathionine-β-synthase
E-selectin: epithelial selectin
Hcy: homocysteine
HFD: high fat diet
HHcy: hyperhomocysteinemia
HS: hidradenitis suppurativa
HBD-2: human beta defensin 2
IBD: inflammatory bowel disease
IFN-γ: interferon gamma
IL-1β: interleukin-1 beta
LPS: lipopolysaccharide
L-selectin: leukocyte selectin
MMP: matrix metalloproteinase
NCSTN: nicastrin
PASH: pyoderma gangrenosum, acne and suppurative hidradenitis
PG: pyoderma gangrenosum
PSEN1: presenilin 1
PSENEN: gamma-secretase subunit
RANTES: regulated on activation normal T-cell expressed and secreted
RT-PCR: real-time polymerase chain reaction
TNF-α: tumor necrosis factor alpha.
